# Medical rehabilitation use and regional bypass among children and adolescents with disabilities: Lessons from South Korea

**DOI:** 10.1371/journal.pone.0360753

**Published:** 2026-10-16

**Authors:** Sungbae Jo

**Affiliations:** 1 Department of Rehabilitation Medicine, Seoul National University Hospital, Seoul, Republic of Korea; 2 Biomedical Research Institute, Seoul National University Hospital, Seoul, Republic of Korea; Ajou University School of Medicine and Graduate School of Medicine, KOREA, REPUBLIC OF

## Abstract

This cross-sectional secondary analysis examined medical rehabilitation use and regional bypass among Korean children and adolescents with registered disabilities, using a two-stage access framework that distinguished entry into medical-institution rehabilitation from out-of-region use after entry. We analyzed public-use data from the 2023 National Survey on Persons with Disabilities, restricting the sample to respondents younger than 19 years (n = 399; weighted national estimate, 88,611). Weighted descriptive statistics and modified Poisson regression with robust standard errors were used to estimate prevalence ratios (PRs) and 95% confidence intervals (CIs). Overall, 50.0% had no medical-institution rehabilitation use, 31.5% used in-region rehabilitation, and 18.6% used out-of-region rehabilitation; among rehabilitation users, 37.1% reported out-of-region care. In stage 1, non-developmental disability type was associated with higher medical rehabilitation use (PR, 1.61; 95% CI, 1.28–2.03), whereas eup/myeon residence was associated with lower use (PR, 0.66; 95% CI, 0.46–0.94). In stage 2, province residence (PR, 2.13; 95% CI, 1.38–3.27) and non-developmental disability type (PR, 1.74; 95% CI, 1.13–2.69) were associated with out-of-region use, while male sex was associated with lower out-of-region use (PR, 0.62; 95% CI, 0.42–0.92). The most common reason for non-use was use of non-medical facilities (27.4%); better treatment and absence of nearby medical institutions were leading reasons for out-of-region care. Pediatric medical rehabilitation access involves challenges to both non-use and out-of-region use, with implications for local entry support, regional capacity-building, and cross-sector service integration.

## Introduction

Rehabilitation is increasingly conceptualized as a functioning-oriented health strategy rather than a service delivered only after acute illness or injury [1]. This perspective is particularly critical in childhood disability, where rehabilitation aims not only to reduce impairment but also to support development, participation, family functioning, and long-term social inclusion [2]. For children and adolescents with disabilities, rehabilitation access is most clinically meaningful when services are available early, locally, and continuously, tailored to their specific developmental needs.

Children and adolescents with disabilities represent a substantial population, and timely, coordinated rehabilitation access is a major health-system concern. Recent comparative analyses of global disability databases estimated that about 291.3 million children and adolescents younger than 20 years have mild-to-severe disabilities, although estimates may differ according to age range, disability threshold, functional domains, and data source [3]. This population is clinically heterogeneous, encompassing motor, sensory, communication, cognitive, behavioral, and adaptive limitations that may require repeated rehabilitation, developmental intervention, assistive technology, caregiver support, and coordination across medical, educational, and community systems [4,5]. Early rehabilitation is important because childhood represents a period of heightened neurodevelopmental plasticity, and evidence-based pediatric rehabilitation guidelines emphasize early diagnosis followed by early, condition-specific intervention [6,7]. Rehabilitation also needs to be geographically accessible because many children require repeated visits, longitudinal monitoring, and caregiver coaching; distance, transportation burden, and uneven local service availability are consistently reported barriers to pediatric rehabilitation access [4]. Therefore, the public health relevance of pediatric disability is not only its prevalence, but also the extent to which health systems can provide timely, appropriate, and geographically accessible rehabilitation across childhood and adolescence.

South Korea provides a policy-relevant setting for examining pediatric rehabilitation access because universal health insurance and a national disability registration system offer formal mechanisms for identifying and supporting children with disabilities, yet formal coverage does not necessarily translate into timely or geographically equitable rehabilitation access [8,9]. This question has added importance in Korea’s exceptionally low-fertility context, where the total fertility rate reached 0.72 in 2023 and the sustainability of pediatric care infrastructure has become a growing concern [10–12]. Previous Korean studies have demonstrated geographic concentration of physical and occupational rehabilitation treatment in the Seoul Capital Area and other large urban centers, as well as high medical service utilization among children with physical or brain disabilities, but have not fully addressed medical rehabilitation entry and out-of-region utilization across the broader pediatric disability population [13,14]. Examining these two stages can clarify whether children with disabilities are unable to enter medical rehabilitation locally or, after entry, must bypass their residential region to obtain care.

Access to rehabilitation is better conceptualized as a pathway than as a single utilization endpoint. Established access frameworks emphasize that care depends not only on whether a service was used, but also on whether individuals can perceive need, seek care, reach services, obtain care, and receive appropriate care [15,16]. Andersen’s behavioral model similarly suggests that predisposing characteristics, enabling resources, and need factors may influence different points in the utilization pathway [17]. This distinction is particularly relevant for pediatric medical rehabilitation, because failure to enter medical-institution rehabilitation and out-of-region use after entry may reflect different mechanisms: the former may indicate unmet need, service substitution, or local entry barriers, whereas the latter may indicate regional bypass due to limited local availability, long waiting times, or perceived differences in service quality.

The 2023 National Survey on Persons with Disabilities is well suited to examine this pathway because it separately assessed medical-institution rehabilitation use, reasons for non-use, inpatient and outpatient rehabilitation, and out-of-region utilization among children and adolescents aged 18 years or younger [9]. Using these data, this study aimed to describe the distribution of pediatric medical rehabilitation access states among Korean children and adolescents with registered disabilities and to identify factors associated with two sequential outcomes: entry into medical-institution rehabilitation and out-of-region utilization among rehabilitation users. We further examined reported reasons for non-use and out-of-region care to help interpret whether observed disparities reflected non-entry into care, service substitution, or regional bypass after entry.

## Methods

### Study design and population

This cross-sectional secondary analysis used public-use unidentified data from the 2023 National Survey on Persons with Disabilities, a nationwide survey commissioned by the Ministry of Health and Welfare and conducted by the Korea Institute for Health and Social Affairs; the public-use microdata were accessed on April 10, 2026 [9]. The survey sampled community-dwelling registered persons with disabilities from the national disability registration database as of May 2023. It used a stratified two-stage cluster sampling design across 17 provinces and metropolitan cities; 285 rural (in Korean, eup/myeon) and urban (in Korean, dong) units were selected, and 8,000 registered persons with disabilities completed structured home-visit interviews. The survey was designed to produce nationally representative estimates for community-dwelling persons with registered disabilities in South Korea.

The analytic population was restricted to respondents younger than 19 years because the child-specific medical rehabilitation items were administered only to this age group. With the final survey weights, the 399 pediatric respondents represented an estimated 88,611 community-dwelling children and adolescents with registered disabilities aged 18 years or younger nationwide. Proxy responses were retained because many children were too young or unable to respond directly.

### Outcomes and covariates

The analysis used a two-stage access framework. The stage-1 outcome was derived from a yes/no item asking whether the child had any experience of rehabilitation treatment at a medical institution. Among respondents reporting such experience, separate yes/no follow-up items assessed inpatient and outpatient rehabilitation and, for each service type used, whether care had been received at a medical institution outside the respondent’s region. Out-of-region care referred to rehabilitation received outside the respondent’s residential region, using the questionnaire’s classification of South Korea into nine geographic regions: the Capital Area, Gangwon, Gyeongbuk, Gyeongnam, Chungbuk, Chungnam (including Daejeon and Sejong), Jeonbuk, Jeonnam, and Jeju. The stage-2 outcome was coded positive when either the inpatient or outpatient out-of-region item was answered affirmatively. No fixed recall period was specified; the items therefore captured any prior experience reported up to the survey date. For descriptive analyses, children were classified into three access states: no medical rehabilitation, in-region use, or out-of-region use.

Covariates were selected a priori based on clinical relevance and data availability. Age was categorized as 0–5, 6–12, and 13–18 years. Sex was coded as female or male. Household income was categorized into weighted quartiles. Registered disability severity was classified as severe or not severe. For descriptive presentation, primary disability type was grouped into six categories: intellectual disability, autism spectrum disability, physical or brain lesion disability, sensory disability, speech/language disability, and internal organ, epilepsy, or other rare disabilities. Because of sparse pediatric cells, regression models used a prespecified analytic grouping: developmental disabilities, defined as speech/language, intellectual, or autism spectrum disabilities, versus all other disability types.

Residential geography was defined in two ways. Local residential setting was classified as urban (dong) versus rural (eup/myeon). Broader administrative region was classified as metropolitan city/Sejong versus province. This administrative classification was distinct from the nine geographic areas used to define the out-of-region outcome.

### Statistical analysis

We used unweighted counts and survey-weighted percentages to summarize sample characteristics and rehabilitation access patterns. Descriptive group comparisons used Rao–Scott tests with final weights only because primary sampling unit and stratum identifiers were not available in the public-use file [18,19]. These *p*-values were interpreted as descriptive, unadjusted comparisons and were not used for covariate selection. Because the public-use file did not provide these design identifiers, clustering and stratification could not be explicitly incorporated into variance estimation. The supplied final weights were retained for weighted point estimation, so the missing design information is expected to affect standard errors, confidence intervals, and p-values more directly than point estimates. Ignoring within-PSU correlation may underestimate standard errors, whereas failing to account for stratification may forgo design-related precision gains and thereby overestimate standard errors. Therefore, the net direction of variance bias cannot be determined.

Adjusted analyses used modified Poisson regression with robust sandwich standard errors to estimate prevalence ratios (PRs) and 95% confidence intervals (CIs) [20]. The stage-1 model was fitted in the full pediatric sample and included age group, sex, analytic disability group, household income quartile, registered disability severity, and local residential setting. The stage-2 model was restricted to rehabilitation users and included age group, sex, analytic disability group, household income quartile, registered disability severity, and broader region. Sensitivity analyses and model diagnostics are summarized in the Supplementary Materials. In an additional analysis, both local residential setting and broader administrative region were included in each model, with the other covariates and estimation method unchanged. Reporting followed STROBE guidance for observational studies [21]. All analyses were conducted in R version 4.5.0 (R Foundation for Statistical Computing, Vienna, Austria), using the survey package for Rao–Scott tests and the sandwich package for HC0 robust variance estimation in weighted modified Poisson models.

### Ethics

This study analyzed de-identified secondary microdata obtained from a national survey. Access to the data was granted following prior approval from the Health and Welfare Data Portal of the Korea Institute for Health and Social Affairs. The study protocol was reviewed and exempted from the ethical review by the Institutional Review Board of Seoul National University Hospital (Approval No. E-2604-161-1736). Because the study utilized secondary data that were fully anonymized prior to access, the requirement for informed consent was waived.

## Results

The pediatric analytic sample included 399 children and adolescents, corresponding to a weighted national estimate of 88,611 (Table 1). Overall, 51.2% were aged 6–12 years, 39.4% were aged 13–18 years, and 66.4% were male. In the detailed primary disability classification, intellectual disability accounted for 45.8% of the weighted sample, followed by autism spectrum disability (25.0%), physical or brain lesion disability (14.5%), sensory disability (6.8%), speech/language disability (6.0%), and internal organ, epilepsy, or other rare disabilities (1.9%). In the analytic grouping used for regression, 76.8% were classified as having developmental disabilities. Most children were classified as having severe disability (87.2%); 21.4% lived in eup/myeon areas and 61.9% lived in provinces.

**Table 1:** Baseline characteristics of the analytic sample according to medical-institution rehabilitation use.

Characteristic	Overall (n = 399)	No Rehabilitation Use (n = 190)	Rehabilitation Use (n = 209)	*p*-value^a^
**Age group, years**				0.066
0–5	38 (9.4)	13 (5.5)	25 (13.4)	
6–12	220 (51.2)	104 (51.7)	116 (50.7)	
13–18	141 (39.4)	73 (42.8)	68 (36.0)	
**Sex**				0.144
Male	278 (66.4)	138 (70.7)	140 (62.1)	
Female	121 (33.6)	52 (29.3)	69 (37.9)	
**Detailed primary disability type**				<0.001
Intellectual disability	112 (45.8)	70 (58.8)	42 (32.8)	
Autism spectrum disability	148 (25.0)	68 (23.0)	80 (27.0)	
Physical or brain lesion disability	37 (14.5)	2 (1.9)	35 (27.0)	
Sensory disability (visual/hearing)	18 (6.8)	8 (7.8)	10 (5.7)	
Speech or language disability	56 (6.0)	24 (5.5)	32 (6.5)	
Internal organ/epilepsy/other rare	28 (1.9)	18 (2.9)	10 (1.0)	
**Analytic disability group**				<0.001
Developmental^b^	316 (76.8)	162 (87.3)	154 (66.3)	
Other disability types	83 (23.2)	28 (12.7)	55 (33.7)	
**Disability severity**				0.370
Severe	337 (87.2)	161 (89.2)	176 (85.2)	
Not severe	62 (12.8)	29 (10.8)	33 (14.8)	
**Household income quartile**				0.194
Q1 (Lowest)	97 (25.4)	51 (26.3)	46 (24.4)	
Q2	110 (27.7)	55 (31.1)	55 (24.4)	
	94 (22.3)	43 (23.5)	51 (21.2)	
Q4 (Highest)	98 (24.6)	41 (19.2)	57 (30.1)	
**Local residential setting**				0.007
Dong (Urban)	323 (78.6)	146 (71.9)	177 (85.3)	
Eup/Myeon (Rural)	76 (21.4)	44 (28.1)	32 (14.7)	
**Broad region**				0.235
Metropolitan	189 (38.1)	84 (34.7)	105 (41.4)	
Province	210 (61.9)	106 (65.3)	104 (58.6)	
**Response type**				0.406
Self response	25 (5.6)	13 (6.6)	12 (4.5)	
Parent proxy	364 (92.1)	174 (92.0)	190 (92.2)	
Non-parent proxy	10 (2.3)	3 (1.4)	7 (3.2)	

**Note:** Data are presented as unweighted number (weighted percentage) unless otherwise indicated. Percentages may not sum to 100% due to rounding.

a *p*-values were calculated using survey-weighted Rao–Scott tests with final survey weights only. Because primary sampling unit and stratum identifiers were not available in the public-use file, *p*-values should be interpreted as descriptive, unadjusted comparisons.

b Developmental disability includes language, intellectual, and autism spectrum disabilities.

In the overall weighted sample, 50.0% had no medical-institution rehabilitation use, 31.5% used in-region rehabilitation, and 18.6% used out-of-region rehabilitation (Fig 1). Among children who had entered medical-institution rehabilitation, 37.1% reported at least one form of out-of-region use. Among rehabilitation users, 22.9% had inpatient rehabilitation experience and 96.1% had outpatient rehabilitation experience; out-of-region care was reported by 47.9% of inpatient users and 35.0% of outpatient users.

**Fig 1:**
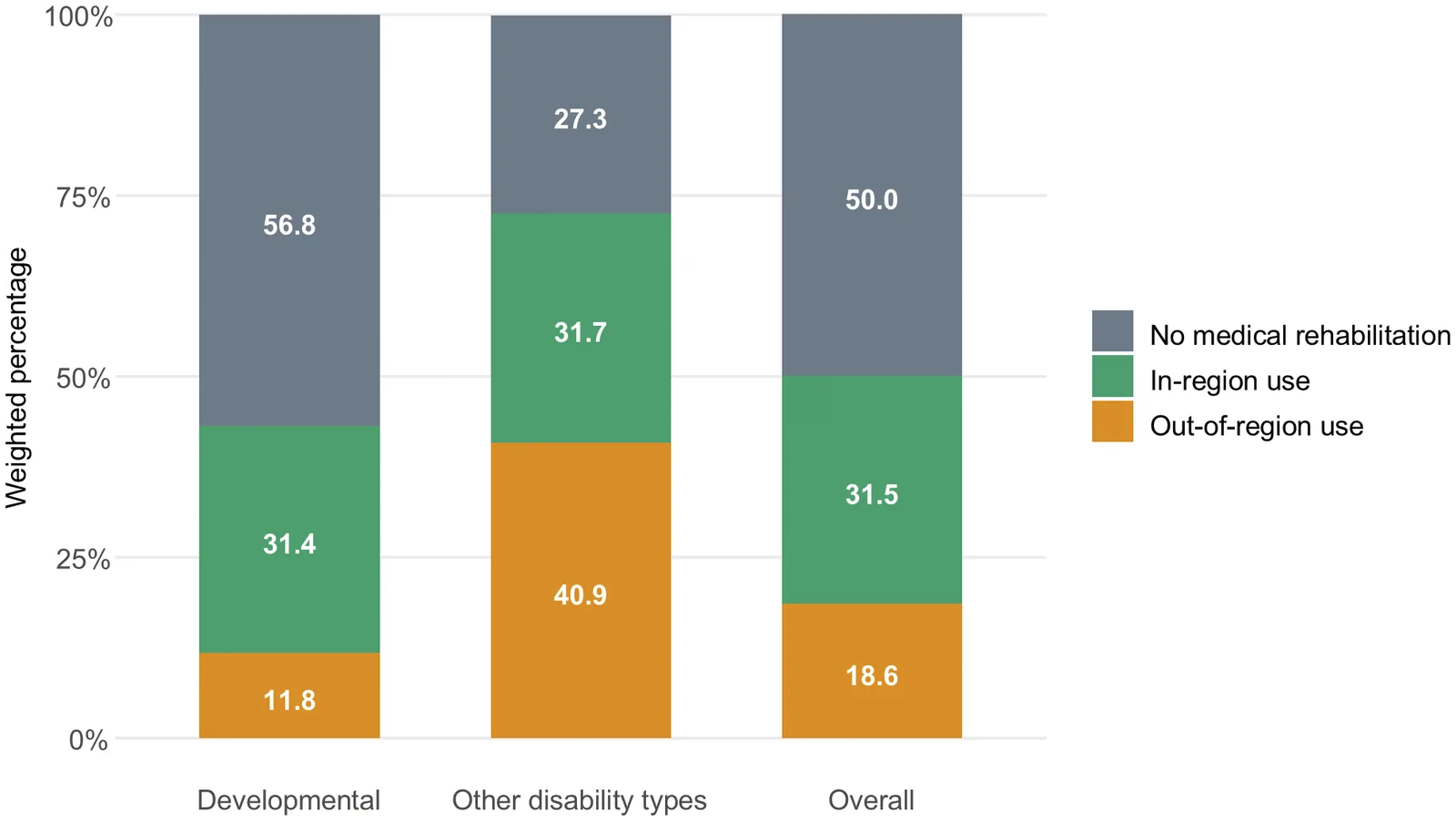
Weighted distribution of medical-institution rehabilitation access states by disability group.

The three-state access distribution differed by disability group and geography. Children with developmental disabilities had higher non-use and lower out-of-region use: 56.8% had no medical-institution rehabilitation, 31.4% used in-region services, and 11.8% used out-of-region services. In contrast, children with other disability types had lower non-use but higher out-of-region use: 27.3% had no medical-institution rehabilitation, 31.7% used in-region services, and 40.9% used out-of-region services. Children living in rural (eup/myeon) areas had higher non-use than those living in urban (dong) areas (65.6% vs. 45.7%). Children living in provinces had lower in-region use and higher out-of-region use than those living in metropolitan areas: in-region use was 25.1% in provinces and 41.8% in metropolitan areas, whereas out-of-region use was 22.2% and 12.6%, respectively (Table 2; Fig 2).

**Table 2:** Weighted distribution of rehabilitation access states among children and adolescents with disabilities.

Characteristic	Unweighted N	Weighted N	No medical rehabilitation, weighted %	In-region use, weighted %	Out-of-region use, weighted %	*p*-value^a^
**Overall**	399	88,611	50.0	31.5	18.6	—
**Disability group**						<0.001
Developmental^b^	316	68,066	56.8	31.4	11.8	
Other disability types	83	20,545	27.3	31.7	40.9	
**Local residential setting**						0.017
Urban (Dong)	323	69,631	45.7	34.8	19.5	
Rural (Eup/Myeon)	76	18,980	65.6	19.1	15.2	
**Broad region**						0.004
Metropolitan	189	33,753	45.6	41.8	12.6	
Province	210	54,858	52.7	25.1	22.2	

**Note:** Percentages use survey weights (WG) and sum to 100% horizontally within each row. Out-of-region use was defined as experiencing either out-of-region inpatient or outpatient medical rehabilitation. Weighted N represents the estimated national population size and is rounded to the nearest whole number.

a Global *p*-values are descriptive, unadjusted survey-weighted Rao-Scott tests with final survey weights only comparing the 3-level access-state distribution within each subgroup. Because primary sampling unit and stratum identifiers were not available, tests used final survey weights only.

b Developmental disability includes language, intellectual, and autism spectrum disabilities.

**Fig 2:**
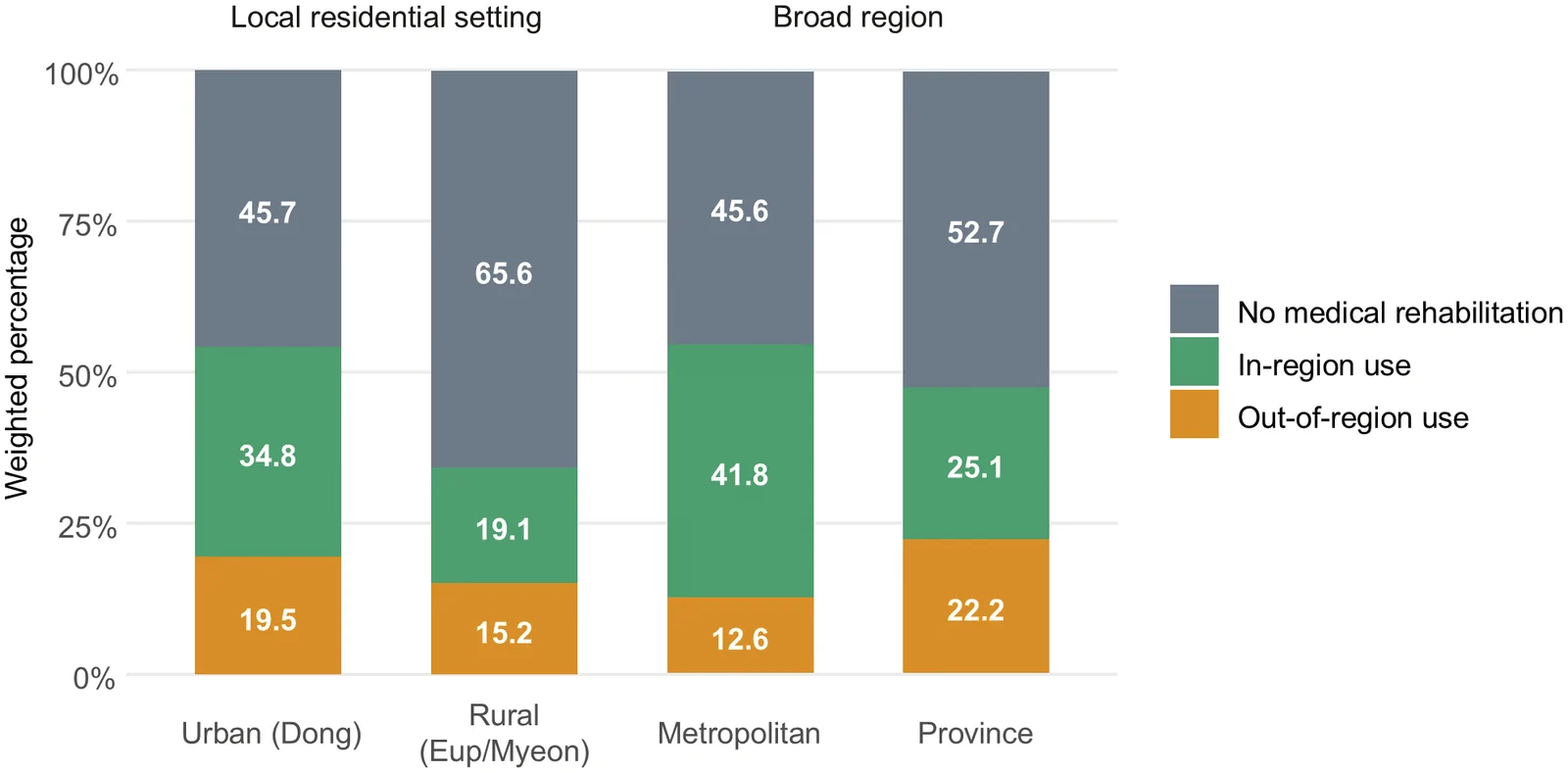
Weighted distribution of medical-institution rehabilitation access states by residential geography.

In the stage-1 multivariable model, children with non-developmental disability types were more likely to use medical-institution rehabilitation than children with developmental disabilities (PR, 1.61; 95% CI, 1.28–2.03; *p* < 0.001). Rural (Eup/myeon) residence was independently associated with lower rehabilitation use compared with urban (dong) residence (PR, 0.66; 95% CI, 0.46–0.94; *p* = 0.022). Age 13–18 years showed a borderline lower prevalence of use compared with age 0–5 years (PR, 0.73; 95% CI, 0.54–0.98; *p* = 0.039). Sex, household income quartile, and registered severity were not significantly associated with stage-1 use.

In the stage-2 model among rehabilitation users, province residence was associated with higher out-of-region utilization compared with metropolitan residence (PR, 2.13; 95% CI, 1.38–3.27; *p* < 0.001). Non-developmental disability type was also associated with higher out-of-region use (PR, 1.74; 95% CI, 1.13–2.69; *p* = 0.012). Male sex was associated with lower out-of-region use than female sex (PR, 0.62; 95% CI, 0.42–0.92; *p* = 0.018). Age group, household income quartile, and registered severity were not independently associated with stage-2 out-of-region use (Table 3; Fig 3).

**Table 3:** Factors associated with entry into medical-institution rehabilitation (Stage 1) and out-of-region use (Stage 2).

Predictor	Stage 1: Any medical-institution rehabilitation use	*p*-value	Stage 2: Out-of-region use among rehabilitation users	*p*-value
**Age group, years**				
0–5 — ref	–	–	–	–
6–12	0.85 (0.63 to 1.13)	0.254	0.90 (0.52 to 1.54)	0.700
13–18	0.73 (0.54 to 0.98)	0.039	0.70 (0.40 to 1.21)	0.202
**Sex**				
Female — ref	–	–	–	–
Male	0.94 (0.74 to 1.18)	0.575	0.62 (0.42 to 0.92)	0.018
**Analytic disability group**				
Developmental — ref	–	–	–	–
Other disability types	1.61 (1.28 to 2.03)	<0.001	1.74 (1.13 to 2.69)	0.012
**Household income quartile**				
Q1 (Lowest) — ref	–	–	–	–
Q2	0.94 (0.67 to 1.32)	0.715	0.97 (0.54 to 1.74)	0.914
Q3	0.98 (0.69 to 1.37)	0.887	1.05 (0.57 to 1.90)	0.884
Q4 (Highest)	1.15 (0.86 to 1.55)	0.338	1.26 (0.72 to 2.20)	0.413
**Disability severity**				
Not severe — ref	–	–	–	–
Severe	1.09 (0.81 to 1.47)	0.557	1.08 (0.67 to 1.73)	0.754
**Local residential setting**				
Dong (Urban) — ref	–	–	–	–
Eup/Myeon (Rural)	0.66 (0.46 to 0.94)	0.022		
**Broad region**				
Metropolitan — ref	–	–	–	–
Province	–	–	2.13 (1.38 to 3.27)	<0.001

**Note:** PR = prevalence ratio; CI = confidence interval. Models used normalized weight WS with HC0 robust sandwich standard errors. Modified Poisson regression with robust variance was used to estimate prevalence ratios for binary outcomes. Developmental disability includes language, intellectual, and autism spectrum disabilities.

**Fig 3:**
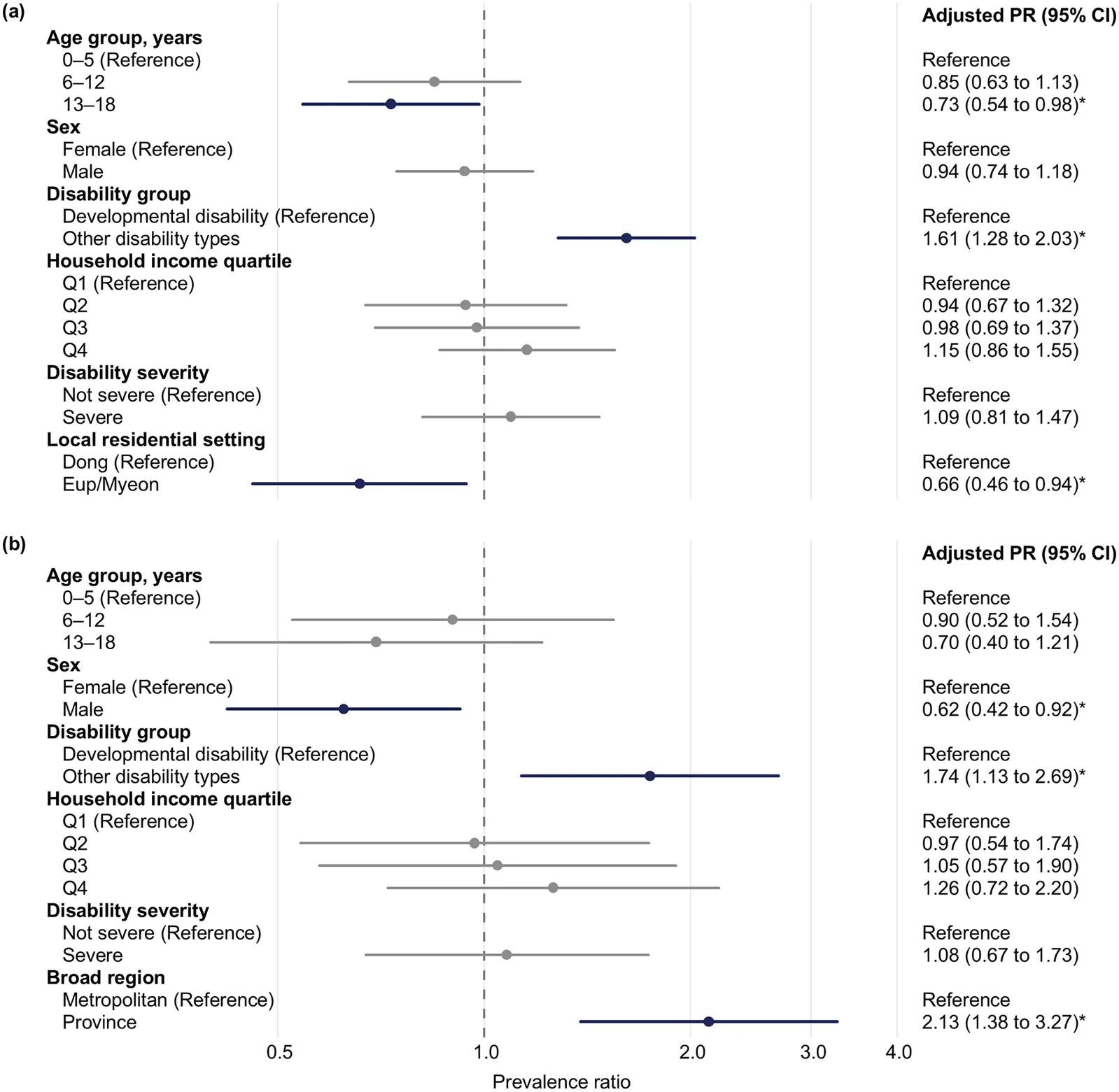
Predictors of medical rehabilitation entry and out-of-region use. Adjusted prevalence ratios and 95% confidence intervals from multivariable modified Poisson regression models for (a) entry into medical rehabilitation (Stage 1) and (b) out-of-region use among rehabilitation users (Stage 2). *p < 0.05.

Among children who did not use medical-institution rehabilitation, the most common reason was use of non-medical facilities (27.4%), followed by no perceived need for rehabilitation (21.6%), no nearby medical institution (17.4%), and cost burden (15.5%)(Supplementary Table S1 in S1 File). Among children receiving out-of-region outpatient rehabilitation, the leading reasons were better treatment (67.8%) and no nearby medical institution (22.8%). For out-of-region inpatient rehabilitation, the dominant reasons were better treatment (60.8%) and no nearby medical institution (39.2%)(Supplementary Table S2–3 in S1 File).

Sensitivity analyses replacing registered severity with functional support need, using a 3-category disability grouping, and restricting the sample to self-respondents and parent proxies yielded estimates broadly consistent with the primary models (Supplementary Table S4 in S1 File). All primary models converged, and multicollinearity diagnostics did not suggest material collinearity (Supplementary Table S5 in S1 File). Stage-specific unadjusted tests are presented in Supplementary Table S6 in S1 File. When both geographic measures were included in each model, eup/myeon residence remained associated with lower stage-1 rehabilitation use (PR, 0.67; 95% CI, 0.47–0.96; *p* = 0.030), and province residence remained associated with higher stage-2 out-of-region use (PR, 2.11; 95% CI, 1.36–3.26; *p* < 0.001). Province residence in stage 1 (PR, 0.93; 95% CI, 0.75–1.15; *p* = 0.487) and eup/myeon residence in stage 2 (PR, 1.11; 95% CI, 0.68–1.81; *p* = 0.677) were not significantly associated with the respective outcomes (Supplementary Table S7 in S1 File)

## Discussion

This nationwide secondary analysis indicates that pediatric medical-institution rehabilitation access among Korean children and adolescents with registered disabilities is best understood as a two-stage pathway: entry into medical-institution rehabilitation and out-of-region use after entry. Half of the weighted pediatric sample had no medical-institution rehabilitation use, whereas more than one-third of rehabilitation users reported out-of-region inpatient or outpatient rehabilitation. These patterns represent different access problems. Non-entry may reflect unmet need, service substitution, perceived lack of need, or local barriers to initiating care, whereas out-of-region use may represent regional bypass after families have already entered the medical rehabilitation system. This interpretation is consistent with access frameworks that define access as a sequence of perceiving need, seeking care, reaching services, obtaining care, and receiving appropriate care, rather than as service use alone [15,16].

The strongest associations were related to disability type and residential geography. Children with non-developmental disability types were more likely than those with developmental disabilities to enter medical-institution rehabilitation and, among users, to receive out-of-region care. Children living in rural (eup/myeon) areas were less likely to enter medical rehabilitation, whereas children living in provinces were more likely to use out-of-region services after entry. These associations persisted when both geographic measures were included in each model. However, the models assessed different outcomes in different analytic samples and did not directly test whether geographic associations differed between stages. These findings support prior Korean claims-based evidence showing that physical and occupational rehabilitation treatment is concentrated in the Seoul Capital Area and other large urban centers [13].

The geographic pattern is consistent with broader literature showing that distance, transportation burden, and uneven service distribution can delay or prevent care [4,22]. These barriers may be especially consequential in pediatric rehabilitation because treatment often requires repeated visits, longitudinal monitoring, and caregiver participation. Studies of children with medical complexity have shown that transportation challenges affect health care access, community participation, safety, and family well-being [23]. Therefore, out-of-region rehabilitation should not be interpreted simply as higher utilization or preference. In this study, the leading reasons for out-of-region care were better treatment and the absence of nearby medical institutions, suggesting that regional bypass reflects perceived quality gaps, local service shortage, or limited pediatric specialization.

The disability-type contrast also requires careful interpretation. Higher non-use among children with developmental disabilities does not necessarily indicate absence of intervention. The leading reason for non-use of medical-institution rehabilitation was use of non-medical facilities, particularly among children with developmental disabilities. Many children with speech/language, intellectual, or autism spectrum disabilities may receive services through welfare, voucher-based, private, or education-linked pathways rather than medical institutions. Thus, the key issue may be fragmentation between medical rehabilitation and non-medical developmental service systems. This interpretation is consistent with evidence that children with developmental disabilities and special health care needs often require coordinated therapy, communication aids, specialty care, educational supports, and family services [5,24].

In contrast, children with non-developmental disability types, especially those with physical or brain lesion disabilities in sensitivity analysis, were more likely to enter medical-institution rehabilitation and to use out-of-region services. This is clinically plausible because these children may require physician-led rehabilitation, physical and occupational therapy, spasticity management, assistive technology, medical monitoring, and multidisciplinary care. Prior Korean pediatric evidence has shown substantial medical service use among children with physical or brain disabilities [14]. Our findings add that these medically oriented rehabilitation pathways may be geographically uneven.

Household income quartile was not independently associated with either stage. This finding should not be interpreted as evidence that financial burden is irrelevant. Cost burden remained a reported reason for non-use, and families may face cumulative expenses for therapy, transportation, caregiving, assistive technology, private services, and parental work disruption. Studies of children with special health care needs have similarly reported financial problems, employment disruption, and difficulty obtaining services [25]. In Korea, universal National Health Insurance, Medical Aid for the lowest-income population, and disability-related public supports may attenuate some direct income-based differences in service use, but these mechanisms do not eliminate financial vulnerability; near-poor National Health Insurance members have been shown to face higher out-of-pocket spending and catastrophic health expenditure than both Medical Aid beneficiaries and higher-income National Health Insurance members [26]. The absence of an independent income gradient in the present analysis therefore suggests that household income quartile alone may not capture the full financial burden of pediatric rehabilitation, and that the dominant disparities observed here were more closely related to geography and disability-specific service pathways than to income alone.

The Korean demographic context strengthens the policy relevance of these results. Korea’s exceptionally low fertility creates a setting in which pediatric services may become increasingly concentrated in larger centers [10,12]. For children with disabilities, who often require repeated and coordinated care over many years, regional maldistribution can have disproportionate life-course consequences. A low-fertility society should not equate a shrinking child population with reduced need for pediatric rehabilitation capacity. Instead, it requires deliberate regional planning for a smaller but high-need population for whom delayed or distant rehabilitation can affect development, participation, and family functioning.

These findings yield several policy implications. Pediatric rehabilitation planning should distinguish between local entry support and regional capacity-building. In rural or underserved municipalities (such as eup/myeon) areas, interventions should prioritize early identification, robust referral linkages, transportation subsidies, outreach assessments, and shared-care pathways to facilitate initial entry into the medical rehabilitation system. At the provincial level, reducing avoidable regional bypass requires strengthening regional pediatric rehabilitation capacity and improving continuity between tertiary centers and local institutions. Furthermore, developmental disability services should be integrated across medical, welfare, education, and family-support systems. Family-centered services are particularly relevant when families must coordinate care across fragmented sectors [27,28]. Given that children with complex needs traverse medical, developmental, behavioral, educational, and social domains, comprehensive care coordination must be formally recognized as a core component of rehabilitation access [29]. Finally, while telerehabilitation and parent coaching may help reduce distance-related barriers, they should be treated as supplements rather than replacements for direct assessment and hands-on intervention [30].

Several limitations should be noted. First, the cross-sectional design precludes causal inference. Second, the public-use file did not include primary sampling unit or stratum identifiers. Therefore, regression models used normalized weights with HC0 robust standard errors, and descriptive *p*-values were obtained using the final weights only. These approaches incorporated the supplied weights but could not reproduce variance estimation under the full complex survey design. Ignoring positive within-cluster correlation may underestimate standard errors, whereas not accounting for stratification may forgo design-related precision and overestimate them. Because the net direction and magnitude of these effects are unknown, confidence intervals and p-values should be interpreted cautiously. This limitation primarily affects uncertainty estimates rather than weighted point estimates. Third, the pediatric sample was modest, requiring disability-type grouping. Fourth, the outcomes measured service experience, not objective clinical need, treatment intensity, quality, wait time, distance, travel cost, or provider supply. Fifth, non-medical facility use was captured as a reason for non-use rather than as a detailed parallel pathway. Finally, the survey included community-dwelling registered persons with disabilities, so findings may not generalize to unregistered children, institutionalized children, or children outside registration criteria.

## Conclusion

In conclusion, pediatric rehabilitation access among Korean children and adolescents with disabilities shows disparities related to geography and disability type. Non-entry is more prevalent in rural areas and among children with developmental disabilities, whereas out-of-region use among rehabilitation users is more frequent among those living in provinces and those with non-developmental disabilities. Addressing these issues requires policies combining local entry support, regional capacity-building, and integrated care coordination to ensure equitable healthcare sustainability amidst Korea’s low-fertility demographic transition.

## Supporting information

S1 File Supplementary methods, results, and S1 to S7 Tables. (DOCX)
