# Supplementary material for: Medical rehabilitation use and regional bypass among children and adolescents with disabilities: Lessons from South Korea
Source: PLoS One. 2026 Oct 16;21(10):e0360753. doi: 10.1371/journal.pone.0360753 (PMC13674712; doi:10.1371/journal.pone.0360753)
Supplement: Supplementary file 1 [file pone.0360753.s001.docx]

**Supplementary Materials**

**Supplementary Methods**

Primary disability type was descriptively grouped into six categories for Table 1, while the main regression models used a binary grouping of developmental disabilities versus other disability types to reduce sparse-cell instability. A sensitivity analysis used three categories: developmental disability, physical or brain lesion disability, and other disability types.

Table 1 *p*-values compared children with and without medical-institution rehabilitation use. Table 2 global *p*-values compared the 3-level access state distribution across subgroups. These tests used survey-weighted Rao–Scott chi-square tests with final weights only because the public-use dataset did not include primary sampling unit or stratum identifiers.

Three sensitivity analyses were performed: replacing registered disability severity with functional support need, using the 3-category disability grouping, and restricting the sample to self-respondents and parent proxies. Multicollinearity was assessed using variance inflation factors calculated from dummy-coded covariate design matrices. Calibration-based goodness-of-fit tests such as the Hosmer–Lemeshow test were not performed because the primary models were modified Poisson regression models for prevalence ratio estimation rather than logistic prediction models.

**Supplementary Results**

Reasons for non-use and out-of-region use are shown in Supplementary Tables S1–S3. Sensitivity analyses are shown in Supplementary Table S4. The main findings were robust: replacing registered severity with support need retained the association between non-developmental disability type and stage-1 use, and between province residence and stage-2 out-of-region use. In the 3-category disability model, physical or brain lesion disability was associated with both higher stage-1 rehabilitation use and higher stage-2 out-of-region use compared with developmental disability. Restricting the sample to self-respondents and parent proxies did not materially change the main estimates.

All primary models converged. Multicollinearity diagnostics showed no evidence of material multicollinearity, with maximum VIFs of 3.20 in the stage-1 model and 2.78 in the stage-2 model (Supplementary Table S5). Stage-specific unadjusted tests are presented in Supplementary Table S6.

**Supplementary Table S1. Reported reasons for non-use of medical-institution rehabilitation among non-users**

| **Variable / Group** | **Reason** | **Unweighted n** | **Weighted %** |
| --- | --- | --- | --- |
| **Overall** |  |  |  |
|  | No need for rehabilitation | 36 | 21.6 |
|  | No nearby medical institution | 35 | 17.4 |
|  | Difficult to visit medical institution | 17 | 7.9 |
|  | Long waiting time | 16 | 10 |
|  | Cost burden | 28 | 15.5 |
|  | Using non-medical facility | 57 | 27.4 |
|  | Other | 1 | 0.2 |
| **Disability group** |  |  |  |
| *Developmental (language/intellectual/autism)* | | | |
|  | No need for rehabilitation | 23 | 17.4 |
|  | No nearby medical institution | 32 | 19.3 |
|  | Difficult to visit medical institution | 14 | 7.2 |
|  | Long waiting time | 15 | 10.4 |
|  | Cost burden | 24 | 15.9 |
|  | Using non-medical facility | 53 | 29.5 |
|  | Other | 1 | 0.2 |
| *Other disability types* |  |  |  |
|  | No need for rehabilitation | 13 | 50.5 |
|  | No nearby medical institution | 3 | 4.1 |
|  | Difficult to visit medical institution | 3 | 12.3 |
|  | Long waiting time | 1 | 7.4 |
|  | Cost burden | 4 | 12.7 |
|  | Using non-medical facility | 4 | 13 |
|  | Other | 0 | 0 |
| **Local setting** |  |  |  |
| Urban (*Dong)* |  |  |  |
|  | No need for rehabilitation | 28 | 21 |
|  | No nearby medical institution | 23 | 12.5 |
|  | Difficult to visit medical institution | 13 | 8.4 |
|  | Long waiting time | 13 | 10.6 |
|  | Cost burden | 20 | 16.7 |
|  | Using non-medical facility | 48 | 30.7 |
|  | Other | 1 | 0.2 |
| Rural (*Eup/Myeon)* |  |  |  |
|  | No need for rehabilitation | 8 | 23.3 |
|  | No nearby medical institution | 12 | 30 |
|  | Difficult to visit medical institution | 4 | 6.6 |
|  | Long waiting time | 3 | 8.6 |
|  | Cost burden | 8 | 12.4 |
|  | Using non-medical facility | 9 | 19.1 |
|  | Other | 0 | 0 |

Note. Percentages are weighted using WG and sum to 100% within each grouping.

**Supplementary Table S2. Reported reasons for out-of-region outpatient rehabilitation among out-of-region outpatient users**

| **Variable / Group** | **Reason** | **Unweighted n** | **Weighted %** |
| --- | --- | --- | --- |
| **Overall** |  |  |  |
|  | No nearby medical institution | 12 | 22.8 |
|  | Better treatment | 45 | 67.8 |
|  | Long wait in-region | 6 | 5.3 |
|  | Lower cost | 0 | 0 |
|  | Out-of-region institution is closer | 4 | 4 |
|  | Other | 0 | 0 |
| **Broad region** |  |  |  |
| *Metropolitan* |  |  |  |
|  | No nearby medical institution | 2 | 9.8 |
|  | Better treatment | 18 | 75.4 |
|  | Long wait in-region | 3 | 9.2 |
|  | Lower cost | 0 | 0 |
|  | Out-of-region institution is closer | 1 | 5.6 |
|  | Other | 0 | 0 |
| *Province* |  |  |  |
|  | No nearby medical institution | 10 | 27.7 |
|  | Better treatment | 27 | 65 |
|  | Long wait in-region | 3 | 3.9 |
|  | Lower cost | 0 | 0 |
|  | Out-of-region institution is closer | 3 | 3.4 |
|  | Other | 0 | 0 |

Note. Percentages are weighted using WG and sum to 100% within each grouping.

**Supplementary Table S3. Reported reasons for out-of-region inpatient rehabilitation among out-of-region inpatient users**

| **Variable / Group** | **Reason** | **Unweighted n** | **Weighted %** |
| --- | --- | --- | --- |
| **Overall** |  |  |  |
|  | No nearby medical institution | 6 | 39.2 |
|  | Better treatment | 13 | 60.8 |
|  | Long wait in-region | 0 | 0 |
|  | Lower cost | 0 | 0 |
|  | Out-of-region institution is closer | 0 | 0 |
|  | Other | 0 | 0 |
| **Broad region** |  |  |  |
| *Metropolitan* |  |  |  |
|  | No nearby medical institution | 1 | 24.3 |
|  | Better treatment | 6 | 75.7 |
|  | Long wait in-region | 0 | 0 |
|  | Lower cost | 0 | 0 |
|  | Out-of-region institution is closer | 0 | 0 |
|  | Other | 0 | 0 |
| *Province* |  |  |  |
|  | No nearby medical institution | 5 | 43.4 |
|  | Better treatment | 7 | 56.6 |
|  | Long wait in-region | 0 | 0 |
|  | Lower cost | 0 | 0 |
|  | Out-of-region institution is closer | 0 | 0 |
|  | Other | 0 | 0 |

Note. Percentages are weighted using WG and sum to 100% within each grouping.

**Supplementary Table S4. Sensitivity models for rehabilitation use and out-of-region use (N=399)**

| **Analysis** | **Predictor** | **PR (95% CI)** | **P-value** |
| --- | --- | --- | --- |
| Stage 1: Any medical-institution rehabilitation use | Other disability types vs developmental disability | 1.61 (1.28 to 2.03) | <0.001 |
|  | Eup/Myeon vs Dong | 0.66 (0.46 to 0.94) | 0.022 |
| Sensitivity 1 stage 1: support need | Other disability types vs developmental disability | 1.76 (1.43 to 2.18) | <0.001 |
|  | Eup/Myeon vs Dong | 0.72 (0.51 to 1.02) | 0.062 |
| Sensitivity 2 stage 1: 3-category disability | Other vs developmental | 0.92 (0.58 to 1.47) | 0.729 |
|  | Physical/brain lesion vs developmental | 2.09 (1.64 to 2.67) | <0.001 |
|  | Eup/Myeon vs Dong | 0.69 (0.49 to 0.98) | 0.037 |
| Sensitivity 3 stage 1: self + parent proxy | Other disability types vs developmental disability | 1.63 (1.29 to 2.06) | <0.001 |
|  | Eup/Myeon vs Dong | 0.67 (0.47 to 0.96) | 0.028 |
| Stage 2: Any out-of-region use among rehabilitation users | Other disability types vs developmental disability | 1.74 (1.13 to 2.69) | 0.012 |
|  | Province vs metropolitan | 2.13 (1.38 to 3.27) | <0.001 |
| Sensitivity 1 stage 2: support need | Other disability types vs developmental disability | 1.67 (1.08 to 2.59) | 0.020 |
|  | Province vs metropolitan | 2.09 (1.35 to 3.22) | <0.001 |
| Sensitivity 2 stage 2: 3-category disability | Other vs developmental | 1.78 (0.94 to 3.40) | 0.079 |
|  | Physical/brain lesion vs developmental | 1.73 (1.07 to 2.80) | 0.025 |
|  | Province vs metropolitan | 2.13 (1.38 to 3.28) | <0.001 |
| Sensitivity 3 stage 2: self + parent proxy | Other disability types vs developmental disability | 1.72 (1.12 to 2.66) | 0.014 |
|  | Province vs metropolitan | 2.14 (1.39 to 3.30) | <0.001 |

Note. PR = prevalence ratio; CI = confidence interval. Stage 1 models evaluate predictors for entering any medical-institution rehabilitation. Stage 2 models evaluate predictors for out-of-region use exclusively among those who successfully entered rehabilitation. *P*-values and PRs were estimated using survey-weighted generalized linear models with robust standard errors to account for the complex survey design.

**Supplementary Table S5. Multicollinearity variance inflation factor statistics**

| **Model / Variable** | **Maximum VIF** | **Mean VIF** |
| --- | --- | --- |
| Stage 1: Any medical-institution rehabilitation use |  |  |
| Age group | 3.2 | 3.16 |
| Sex | 1.05 | 1.05 |
| Disability group | 1.10 | 1.10 |
| Household income quartile | 1.56 | 1.55 |
| Disability severity | 1.04 | 1.04 |
| Local residential setting | 1.04 | 1.04 |
| Stage 2: Any out-of-region use among rehabilitation users |  |  |
| Age group | 2.78 | 2.73 |
| Sex | 1.09 | 1.09 |
| Disability group | 1.14 | 1.14 |
| Household income quartile | 1.68 | 1.66 |
| Disability severity | 1.03 | 1.03 |
| Broad region | 1.05 | 1.05 |

Note. VIF = Variance Inflation Factor. Multicollinearity was assessed for all predictors included in the regression models.

**Supplementary Table S6. Unadjusted survey-weighted tests for access distribution (N=399)**

| **Variable** | **Global P for 3-state access distribution** | **Stage 1 *p* for medical rehabilitation use** | **Stage 2 *p* for out-of-region use among rehabilitation users** |
| --- | --- | --- | --- |
| Disability group | <0.001 | <0.001 | 0.001 |
| Local residential setting | 0.017 | 0.007 | 0.44 |
| Broad region | 0.004 | 0.235 | 0.002 |

Note. *P*-values were derived from unadjusted survey-weighted tests. The global *p*-value indicates overall distributional differences across the three access states (non-use, in-region use, and out-of-region use). Stage 1 *p*-values evaluate differences between users and non-users. Stage 2 *p*-values evaluate differences between in-region and out-of-region care exclusively among rehabilitation users.

**Supplementary Table S7. Adjusted prevalence ratios for medical-institution rehabilitation use and out-of-region use with both geographic measures included in each model.**

| **Predictor** | **Stage 1: Any medical-institution rehabilitation use** | ***p*-value** | **Stage 2: Out-of-region use among rehabilitation users** | ***p-value*** |
| --- | --- | --- | --- | --- |
| **Local residential setting** | | | |  |
| Urban (Dong) — ref | – | – | – | – |
| Rural (Eup/Myeon) | 0.67 (0.47–0.96)^a^ | 0.030 | 1.11 (0.68–1.81) | 0.677 |
| **Broad region** | | | |  |
| Metropolitan — ref | – | – | – | – |
| Province | 0.93 (0.75–1.15) | 0.487 | 2.11 (1.36–3.26) | <0.001 |
| **Age group, years** | | | |  |
| 0–5 — ref | – | – | – | – |
| 6–12 | 0.85 (0.63–1.13) | 0.259 | 0.90 (0.52–1.54) | 0.693 |
| 13–18 | 0.73 (0.54–0.99) | 0.041 | 0.70 (0.40–1.22) | 0.209 |
| **Sex** |  |  |  |  |
| Female — ref | – | – | – | – |
| Male | 0.94 (0.75–1.19) | 0.617 | 0.62 (0.42–0.92) | 0.019 |
| **Disability group** | | | |  |
| Developmental disability^b^ — ref | – | – | – | – |
| Other disability types | 1.61 (1.29–2.02) | <0.001 | 1.74 (1.13–2.67) | 0.012 |
| **Household income quartile** | | | |  |
| Q1 (Lowest) — ref | – | – | – | – |
| Q2 | 0.94 (0.67–1.32) | 0.719 | 0.97 (0.54–1.77) | 0.931 |
| Q3 | 0.99 (0.70–1.39) | 0.942 | 1.07 (0.58–1.95) | 0.838 |
| Q4 (Highest) | 1.16 (0.86–1.55) | 0.333 | 1.29 (0.73–2.29) | 0.386 |
| **Registered disability severity** | | | |  |
| Not severe — ref | – | – | – | – |
| Severe | 1.08 (0.80–1.45) | 0.628 | 1.08 (0.67–1.74) | 0.743 |

Note. PR = prevalence ratio; CI = confidence interval. ^a^PR (95% CI). Stage 1 included 399 children and adolescents, of whom 209 reported medical-institution rehabilitation use. Stage 2 included those 209 users, of whom 74 reported out-of-region use. These sample sizes and event counts are unweighted. PRs were estimated using the normalized survey weight (WS) in modified Poisson regression with HC0 robust standard errors. ^b^Developmental disability includes speech/language, intellectual, and autism spectrum disabilities.
